# Immune recovery uveitis in Whipple’s disease: an unusual ocular presentation

**DOI:** 10.1186/s12348-024-00390-5

**Published:** 2024-02-13

**Authors:** Hippolyte Lequain, Stéphane Abramowicz, Julien Seiller, Amro Abukhashbah, Carole Burillon, Emmanuelle Vignot, Olivier Brunet, Pascal Sève

**Affiliations:** 1 Department of Internal Medicine, Croix-Rousse University Hospital, Hospices Civils de Lyon, 103 Grande-Rue de La Croix-Rousse, 69004 Lyon, France; 2 Department of Rheumatology, Edouard Herriot University Hospital, Hospices Civils de Lyon, Lyon, France; 3 Department of Ophthalmology, Edouard Herriot University Hospital, Hospices Civils de Lyon, Lyon, France; 4 Department of Ophthalmology, King Abdulaziz University, Rabigh, Saudi Arabia; 5 Université Claude Bernard Lyon 1, Research On Healthcare Performance (RESHAPE), INSERM U1290, Lyon, France

**Keywords:** Uveitis, Whipple’s disease, Immune reconstitution inflammatory syndrome, Immune recovery uveitis, Neuroretinitis, Retinal vasculitis

## Abstract

**Purpose:**

To describe an unusual case of Whipple’s disease (WD) complicated by uveitis, and subsequent paradoxical worsening after effective antibiotic treatment targeting *Tropheryma whipplei* (TW).

**Methods:**

Case report.

**Results:**

A 53-year-old male presented with bilateral knee arthritis, weight loss, chronic low-grade fever, and cognitive disorders. He was under treatment with tumor necrosis factor α inhibitors (TNFi) for seronegative spondyloarthritis. Given this unusual clinical presentation, further investigations were performed and revealed blood, saliva, stool, synovial fluid and cerebrospinal fluid positivity for TW, confirming the diagnosis of systemic WD. Ophthalmologic examination revealed bilateral posterior uveitis and an aqueous humor sample confirmed the presence of intraocular TW. TNFi were stopped, and the patient was subsequently treated with adequate antibiotics (ceftriaxone, followed by doxycycline and hydroxychloroquine), and subconjunctival corticosteroid injections. After a transient improvement of the ocular symptoms, he presented a recurrence of posterior segment inflammation, leading to repeated PCR testing for TW which were negative. Therefore, paradoxical worsening of the inflammation in the context of immune recovery uveitis (IRU) was thought to be the culprit. The patient was treated with systemic corticosteroid therapy, allowing for rapid improvement of the ocular findings.

**Conclusions:**

This case underlines the possibility of IRU complicating WD. Ophthalmologists, rheumatologists, and internists should be aware of this rare complication, particularly in the context of previous immunosuppressive therapy.

## Introduction

Whipple's disease (WD) is a rare systemic infection caused by the gram-positive actinobacterium *Tropheryma whipplei *(TW), which typically affects middle-aged white men [1]. Manifestations of WD include weight loss, diarrhea, abdominal pain, chronic seronegative arthritis, and central nervous system (CNS) involvement [1]. Ocular involvement in WD is found in up to 6% of patients, and it is usually found in association with systemic manifestations [2]. Frequent ocular findings include chronic uveitis (vitritis, retinitis, choroiditis, panuveitis), optic neuritis, and optic nerve edema [3, 4]. Ocular surface (crystalline keratopathy, scleral nodules) [5–7], and neuro-ophthalmological involvement (oculomasticatory myorhythmia, supranuclear ophthalmoplegia) is less common [8, 9].

In an unlucky twist, atypical presentations of WD can sometimes mimic rheumatic disease such as spondyloarthritis, and immunosuppressive therapy is therefore sometimes inadequately used in these situations [3, 4, 10, 11]. This can predispose to the development of immune reconstitution inflammatory syndrome (IRIS) when patients are ultimately weaned off immunosuppressive drugs and given adequate antibiotic treatment [1, 10, 12]. IRIS is a paradoxical flare-up of inflammatory signs and symptoms after effective antimicrobial treatment against an infectious agent, in the additional context of reversal of prior immune suppression [13, 14]. WD-associated IRIS (WD-IRIS) occurs in approximately 10% of patients treated with antibiotics, and can present with a wide range of clinical manifestations, such as fever, arthritis, erythema nodosum, and uveitis [1, 15].

We describe the case of a 53-year-old man presenting with WD uveitis and subsequent paradoxical worsening of ocular symptoms after effective antibiotic therapy, revealing a superimposed immune recovery uveitis (IRU) in the context of WD-IRIS.

## Case description

A 53-year-old man with a history of chronic HBV hepatitis was admitted to the rheumatology department in August 2020 for low-back pain, left hip pain, and bilateral knee arthritis. He carried a diagnosis of HLA-B27 negative axial spondyloarthropathy (AS), currently failing treatment with a third sequential tumor necrosis factor-α inhibitor (TNFi). He had previously received treatment with etanercept and adalimumab, and was currently treated with intravenous infliximab.

The diagnosis of HLA-B27 negative AS was made in 2017, on the basis of recurrent bilateral knee arthritis and long-standing low-back pain with inflammatory remodeling on the spine magnetic resonance imaging (MRI). In addition to the arthritis, the patient suffered from an unintentional weight loss of roughly 30 kgs in the last 6 months, a low-grade fever chronically hovering around 38 °C, cognitive disorders with personality changes, and he complained of decreased vision with floaters in both eyes during the past year. The patient did not complain of abdominal pain or diarrhea.

Given the unusual presentation for AS and the ineffectiveness of 3 different TNFi (etanercept, adalimumab, and infliximab), the diagnosis of AS was questioned and further investigations were performed.

Laboratory tests revealed an elevated C-reactive-protein (CRP) level at 60 mg/L, and a prolonged erythrocyte sedimentation rate (ESR) at 84 mm/h. Complete blood count found microcytic anemia, with normal white blood cell and platelet counts. Blood electrolytes, renal and liver function tests were normal. Blood cultures and immunologic assays (anti-cyclic citrullinated peptide antibodies, antinuclear antibodies, and rheumatoid factor) were negative, and multiple infectious diseases were serologically ruled out (*Treponema pallidum*, *Rickettsia spp*, *Coxiella spp, Brucella spp, Bartonella spp,* HIV, Cytomegalovirus, and Epstein-Barr virus). Thoraco-abdominal computed tomography (CT) scan revealed portal hypertension with splenomegaly, possibly in the context of chronic HBV hepatitis.

**Fig. 1:**
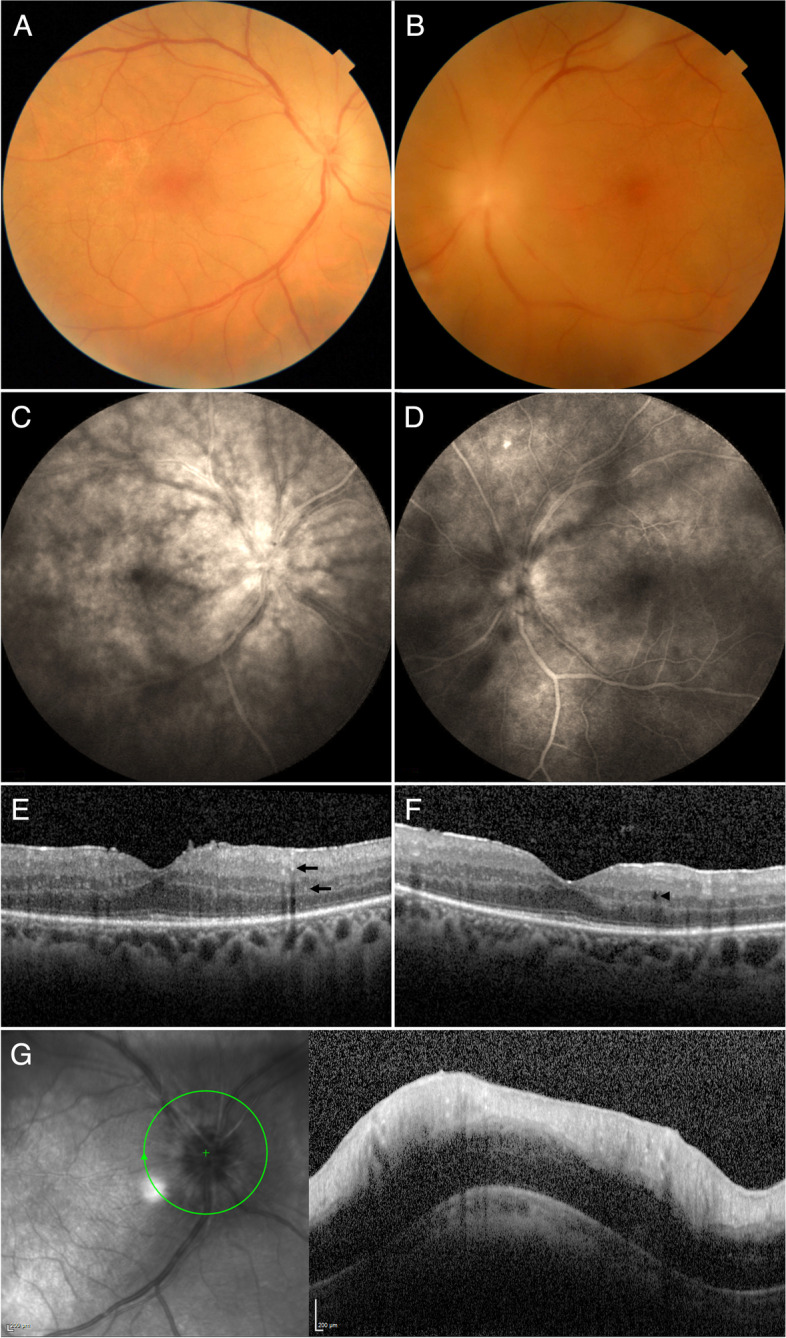
Multimodal imaging of Whipple’s disease uveitis at presentation. **A** Fundus photograph of the right eye revealing minimal vitreous haze associated with severe optic nerve head edema, and mild whitish retinal changes in the temporal macula. **B** Fundus photograph of the left eye at presentation revealing moderate vitreous haze associated with optic nerve head edema with very hazy details. **C** Fluorescein angiography (FA) of the right eye showing profuse optic disc leakage, extensive capillary leakage over the entire posterior pole, and an associated area of venous vasculitis at the level of the superotemporal arcade. **D** FA of the left eye showing moderate optic disc leakage, capillary leakage, and superotemporal venous vasculitis. A small punctiform hyperfluorescent focus can be seen above the disc. **E** Macular spectral-domain optical coherence tomography (SD-OCT) of the right eye demonstrating a mild epiretinal membrane (ERM), diffuse macular thickening without cystic changes, and some discrete intraretinal hyperreflective dots (arrows). **F** Macular SD-OCT of the left eye demonstrating a mild ERM, diffuse macular thickening with one intraretinal cystic cavity (arrowhead), and some discrete intraretinal hyperreflective dots. **G** Optic nerve head SD-OCT of the right eye showing severe disc edema, mainly secondary to retinal nerve fiber layer (RNFL) edema

Ophthalmologic examination found a decreased best-corrected visual acuity (BCVA) of 20/30 in both eyes, normal intraocular pressures (IOP), normal slit-lamp examination (SLE) with no anterior chamber cells or flare, presence of 1 + vitritis in the right eye and 3 + vitritis in the left, as well as bilateral optic nerve head edema (Fig. 1A–B). Some snowballs could be also seen in both eyes. Fluorescein angiogram (FA) revealed leakage in both optic nerve heads and venous vasculitis in both eyes (Fig. 1C–D). Indocyanine green angiography (ICGA) was normal in both eyes. Macular and optic nerve head spectral-domain optical coherence tomography (SD-OCT) found bilateral diffuse macular thickening, faint intraretinal exudates, and severe optic nerve head edema (Fig. 1E–G). A diagnosis of bilateral posterior uveitis with mild neuroretinitis and intense vitreous reaction was made.

Considering these elements, the possibility of WD was raised. PCR tests for TW were ordered, and these were positive from multiple sites (blood, urine, saliva, stool, synovial fluid, CSF, and aqueous humor). PCR of duodenal biopsy specimens were also positive for TW. However, periodic acid-Schiff (PAS)-positive foamy macrophages were not seen. These findings led to revise the diagnosis of HLA-B27 negative AS to a diagnosis of classical WD with bilateral posterior uveitis and vitritis.

**Fig. 2:**
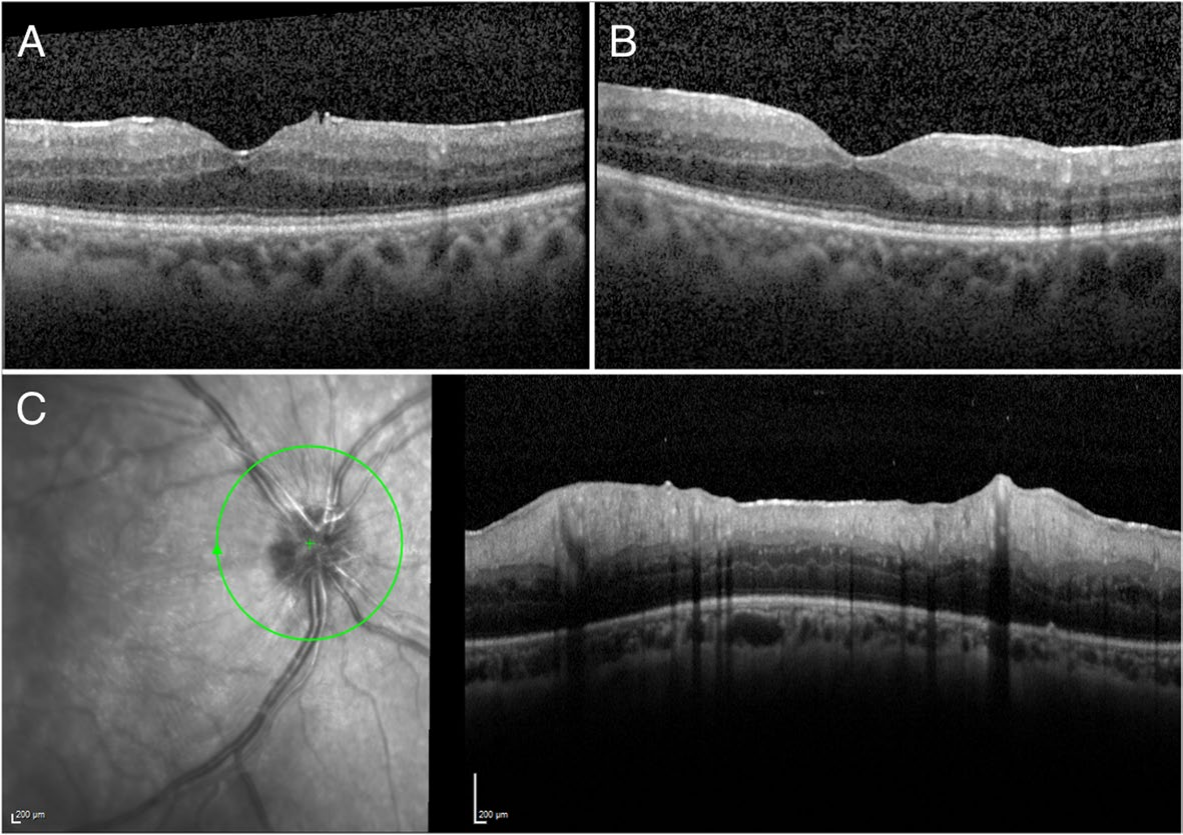
Macular and optic nerve head optical coherence tomography showing initial improvement in anatomical features. **A** Macular spectral-domain optical coherence tomography (SD-OCT) of the right eye demonstrating mild reduction in diffuse macular thickening, and disappearance of the intraretinal hyperreflective dots. **B** Macular SD-OCT of the left eye also showing mild reduction in diffuse macular thickening, and disappearance of the intraretinal cystic cavity. **C** Optic nerve head SD-OCT of the right eye showing notable improvement in disc edema

The patient received treatment with intravenous ceftriaxone 4 g/day for one month, followed by a combination therapy of doxycycline 100 mg twice a day and hydroxychloroquine 200 mg three times a day. Betamethasone subconjunctival injections were also performed to control the uveitis. This resulted in a progressive improvement of fever, joint pain, inflammatory syndrome, and weight gain. The ocular inflammation also improved in both eyes with fewer floaters, reduction of vitritis, and reduction of macular and optic nerve head thicknesses (Fig. 2A–C).

**Fig. 3:**
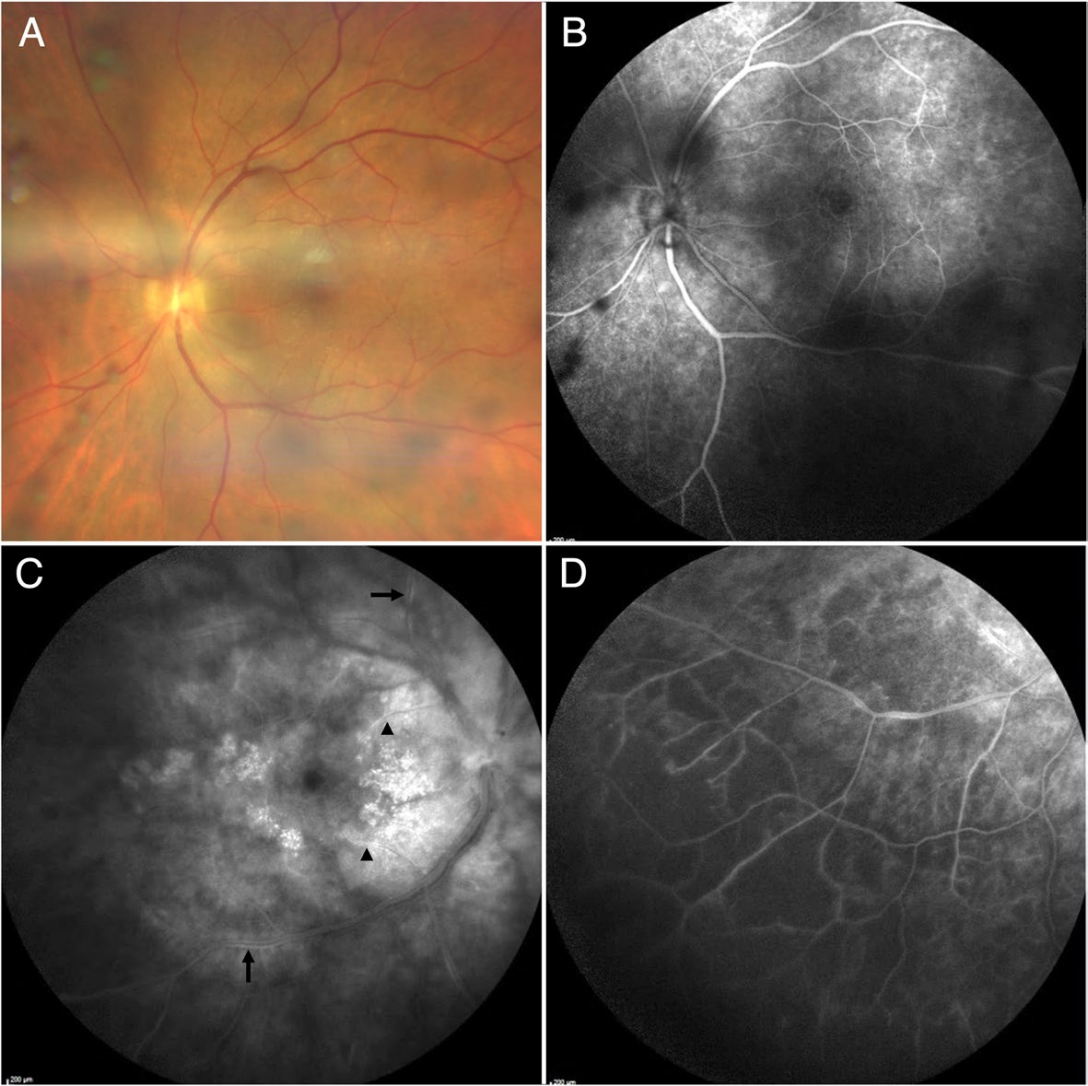
Immune recovery uveitis in Whipple’s disease-associated immune reconstitution inflammatory syndrome. **A** Fundus photograph of the left eye showing mild vitreous haze, vitreous precipitates and mild optic nerve edema. **B** Fluorescein angiography (FA) of the left eye revealing vitreous precipitates and mild capillary leakage. **C** FA of the right eye showing severe optic disc leakage, multifocal segmental arterial (arrowhead) and venous (arrow) vasculitis, severe capillaropathy and grainy hyperfluorescent changes in the macula. **D** FA of the right eye showing severe ischemic changes in the inferior temporal periphery with multifocal areas of occlusive venous vasculitis

Three months after initiating antibiotic treatment for WD, the patient complained of a recurrence of floaters and vision loss. BCVA was 20/30 in the right eye, and 20/32 in the left, IOP and SLE were unremarkable. Fundus examination showed vitreous precipitates (Fig. 3A). FA showed optic disc edema associated with areas of occlusive vasculitis in both eyes (Fig. 3B–D). Macular SD-OCT found recurrence of macular edema in the right eye. Repeat PCR testing for TW in blood, saliva, stool, CSF, and aqueous humor samples were negative.

Presence of an inflammatory recurrence after initial improvement under antibiotic treatment and discontinuation of TNFi, and the absence of another identifiable cause for the inflammatory relapse, led to a diagnosis of WD-IRIS in the form of an IRU. We started treatment with intravenous methylprednisolone (1 mg/kg/day for three consecutive days), followed by an oral prednisone tapering regimen (starting at 1 mg/kg/day) during one year. Combination therapy with doxycycline and hydroxychloroquine was continued in parallel to the corticosteroids, and was given for a total of one year. This allowed improvement of the ocular inflammation, with some relapses however.

Indeed, almost 3 years of follow-up were marked by several ocular inflammatory relapses characterized by intermediate uveitis with snowballs predominantly in the left eye without systemic inflammation or symptoms. Inflammatory relapses were treated with subconjunctival triamcinolone acetonide and intravitreal Ozurdex injections, and were complicated by high intraocular pressure (up to 29 mmHg) in the left eye and cataracts in both eyes.

**Fig. 4:**
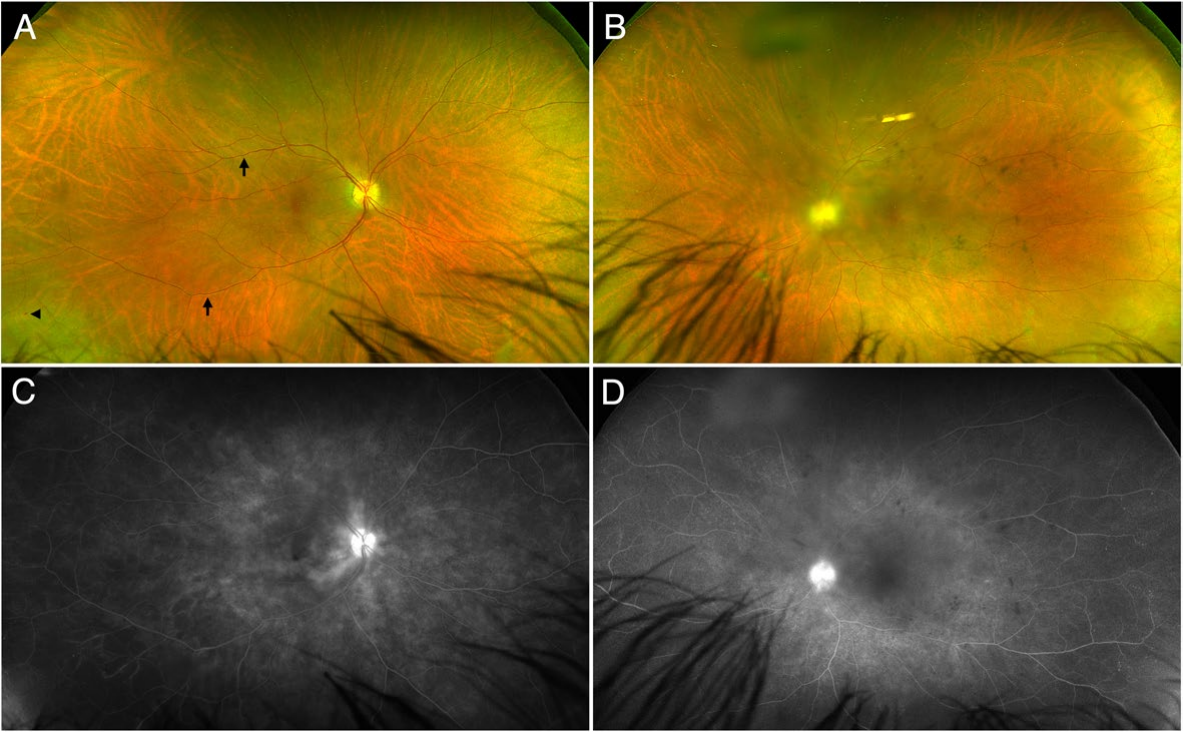
Posterior segment findings after 2-year follow-up of Whipple’s disease uveitis and subsequent immune recovery uveitis. **A** Ultra-wide field (UWF) fundus photograph of the right eye showing discrete temporal venous sheathing (arrows) and a small intraretinal hemorrhage in the inferior temporal periphery (arrowhead). **B** UWF fundus photograph of the left eye showing hazy details because of subcapsular posterior cataract. Vitreous precipitates can be seen in front of the posterior pole and across the inferior periphery. Note areas of proximal venous vascular sheathing. **C** UWF fluorescein angiography (FA) of the right eye showing mild capillaropathy, and ischemic areas in the temporal periphery. **D** UWF FA of the left eye showing multifocal vitreous precipitates, and mild telangiectatic vascular changes in the temporal periphery. No vascular leakage can be seen

At the last follow-up visit in April 2023, the patient had a BCVA of 20/50 in the right eye, and 20/100 in the left due to uveitic and steroid-induced cataracts. There were some chronic inactive snowballs in the left eye (Fig. 4A–B). Fluorescein angiogram revealed absence of optic nerve head edema, and absence of active retinal vasculitis (Fig. 4C–D). Macular SD-OCT showed normal macular thickness in both eyes.

## Discussion

We report a case of bilateral WD uveitis complicated by IRU after discontinuation of TNFi and effective antibiotic treatment for WD. Bilateral IRU was characterized by recurrent posterior segment involvement (snowballs, occlusive vasculitis) with negative repeat TW PCR testing in multiple samples, including aqueous humor samples. Management of posterior segment inflammation was difficult despite systemic, subconjunctival, and intravitreal corticosteroid use and was complicated by high intraocular pressures. In the literature, oral corticosteroid therapy is the recommended therapy of choice for WD-IRIS, and has shown effectiveness against IRU without the need for periocular or intraocular steroids [1].

The initial rheumatologic presentation, in the absence of gastrointestinal involvement, led to misdiagnose WD as an inflammatory rheumatic disease, and to treat the patient with TNFi. According to the literature, a significant percentage of patients receive inappropriate immunosuppressive therapy in WD which can promote complications such as WD-IRIS and TW endocarditis, and may also induce PAS-negative duodenal biopsies [10].

IRIS is an aberrant reconstitution of immunity during normalization of CD4 + T-cell counts, which are insufficiently counterbalanced by regulatory T cells, resulting in a dysregulated immune response and inflammatory flare-ups [1, 10, 15, 16]. About 10% of patients treated with antimicrobials for WD will develop WD-IRIS, with previous immunosuppressive treatment being a major risk factor, and especially in case of TNFi use [10, 15]. Fever and arthritis are the main features of WD-IRIS. However, a wide range of inflammatory manifestations can occur, such as erythema nodosum, meningitis, pleuritis and endocarditis [1].

Ocular manifestations of IRIS may also occur, and literature review focusing on ocular and orbital WD-IRIS identified six patients [12, 17]. In a 15-patient cohort of WD-IRIS, Feurle et al. reported four inflammatory orbitopathies and one uveitis [12]. Schaller et al. presented a case of WD-IRIS with erythema nodosum-like lesions and an orbital pseudotumor [17]. In total, five patients presented with an orbital WD-IRIS and one presented with an IRU. The response to treatment was variable, and inflammatory symptoms regressed after long-term immunosuppressive treatment for most patients (66.6%). However, some patients remain poorly controlled under treatment, or remain steroid-dependent.

In summary, WD-IRIS represents a difficult diagnostic situation, and there is likely a continuum between local ocular manifestations of WD and ocular WD-IRIS manifestations. Nevertheless, as shown in our case, results of repeated PCR testing can guide strategy. If PCR remains positive, previous antibiotic therapy should be considered unsuccessful, prompting the initiation of new antibiotic therapy, possibly with alternative molecules, like trimethoprim/sulfamethoxazole for example. If PCR tests become negative, WD-IRIS should be considered as the likely cause of persistent symptoms, leading to the initiation of corticosteroids and immunosuppressive agents.

## Conclusions

This case underlines the importance of considering WD-IRIS in the context of ocular or orbital inflammatory relapses or persistence, after effective antibiotic treatment in WD. Ophthalmologists, rheumatologists, and internists should be aware of this rare complication.

## Data Availability

All data shared are available upon reasonable request to the corresponding author: Pr. Pascal Sève, MD, PhD; email address: pascal.seve@chu-lyon.fr.
